# Recent Advances in Molecular Docking for the Research and Discovery of Potential Marine Drugs

**DOI:** 10.3390/md18110545

**Published:** 2020-10-30

**Authors:** Guilin Chen, Armel Jackson Seukep, Mingquan Guo

**Affiliations:** 1 Key Laboratory of Plant Germplasm Enhancement & Specialty Agriculture, Wuhan Botanical Garden, Chinese Academy of Sciences, Wuhan 430074, China; glchen@wbgcas.cn (G.C.); seukep.armel@ubuea.cm (A.J.S.); 2 Sino-Africa Joint Research Center, Chinese Academy of Sciences, Wuhan 430074, China; 3 Innovation Academy for Drug Discovery and Development, Chinese Academy of Sciences, Shanghai 201203, China; 4 Department of Biomedical Sciences, Faculty of Health Sciences, University of Buea, P.O. Box 63 Buea, Cameroon

**Keywords:** protein–ligand interaction, molecular docking, marine drugs, target protein, mechanism of action

## Abstract

Marine drugs have long been used and exhibit unique advantages in clinical practices. Among the marine drugs that have been approved by the Food and Drug Administration (FDA), the protein–ligand interactions, such as cytarabine–DNA polymerase, vidarabine–adenylyl cyclase, and eribulin–tubulin complexes, are the important mechanisms of action for their efficacy. However, the complex and multi-targeted components in marine medicinal resources, their bio-active chemical basis, and mechanisms of action have posed huge challenges in the discovery and development of marine drugs so far, which need to be systematically investigated in-depth. Molecular docking could effectively predict the binding mode and binding energy of the protein–ligand complexes and has become a major method of computer-aided drug design (CADD), hence this powerful tool has been widely used in many aspects of the research on marine drugs. This review introduces the basic principles and software of the molecular docking and further summarizes the applications of this method in marine drug discovery and design, including the early virtual screening in the drug discovery stage, drug target discovery, potential mechanisms of action, and the prediction of drug metabolism. In addition, this review would also discuss and prospect the problems of molecular docking, in order to provide more theoretical basis for clinical practices and new marine drug research and development.

## 1. Introduction

**Figure 1:**
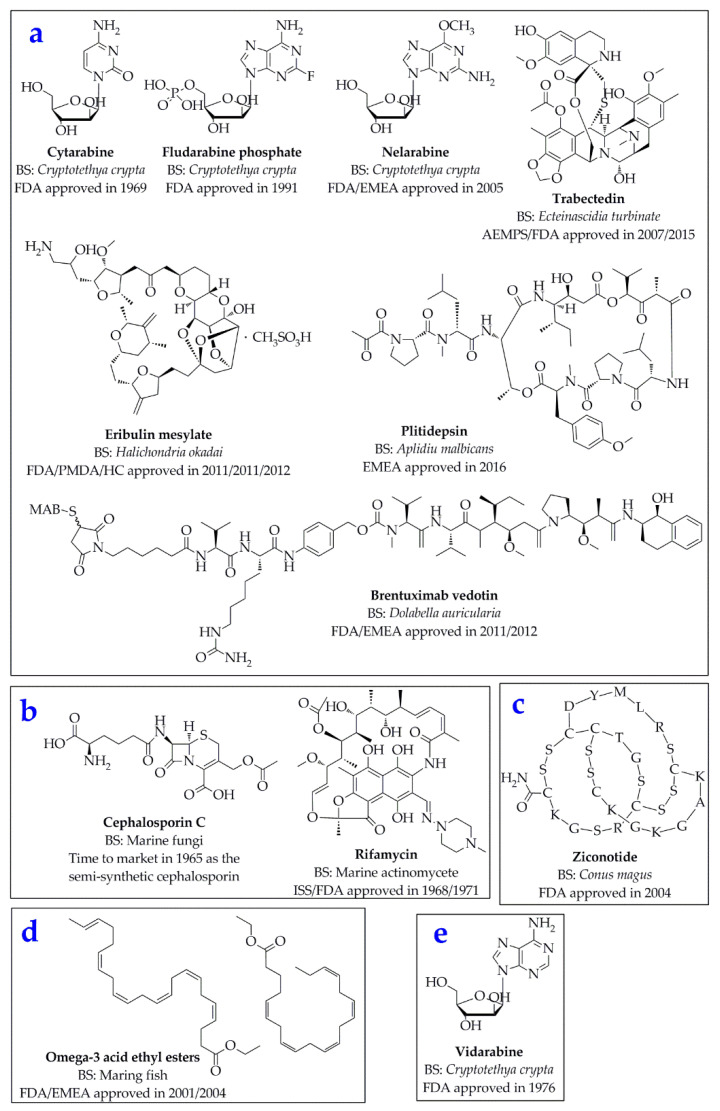
Chemical structures of the 12 approved marine drugs of anticancer (**a**), antibacterial (**b**), analgesic (**c**), cardiovascular (**d**), and antiviral (**e**) agents [3,8]. BS: biological source. Abbreviations of amino acids: A, Alanine; C, Cysteine; D, Aspartic acid; G, Glycine; K, Lysine; L, Leucine; M, Methionine; R, Arginine; S, Serine; T, Threonine; Y, Tyrosine. FDA, Food and Drug Administration (USA); EMEA, European Medicines Evaluation Agency; AEMPS, Agencia Española de Medicamentos y Productos Sanitarios (Spain); HC, Health Canada; ISS, Istituto Superiore di Sanità (Italy); PMDA, Pharmaceuticals and Medical Devices Agency (Japan).

The ocean accounts for about 70% of the area of the Earth, in which numerous marine organisms possess unique and novel components that are not found on land, with specific biological properties of high activities and efficacies. Since the National Cancer Institute (NCI) screened marine resources for anti-cancer activities in 1968, research on marine drugs has entered into an independent field [1]. The abundant marine organisms can produce a variety of natural active substances with novel structures and remarkable activities, and marine drugs developed on this basis play an important role in the fields of anti-inflammatory, anti-tumor, anti-virus, anti-bacteria, anti-coagulation, malaria, analgesia, neurological disorders, cardiovascular, and cerebrovascular diseases [2,3]. By 2015, more than 26,680 compounds had been isolated and identified from marine-derived sources, and the number is still growing at an average of 1000 per year [4]. Since the earliest clinical application of marine drugs—the antibacterial Cephalosporin C [5] from marine fungi and the anti-tuberculosis drug Rifamycin [6,7] from marine Actinomycetes in 1960s—many national and regional drug regulatory agencies have approved several marine natural products and derivative drugs derived from marine organisms (Figure 1). Hence, marine drugs have become one of the frontier areas with the most abundant drug resources, the most complete preservation and the most potential for drug research and development (R&D).

However, the discovery and development of marine drug candidates from the ocean have been hampered from the outset by the difficulties of how to obtain a large number of rare compounds for research. The key to the clinical effect of the drugs is that their bioactive components bind to the corresponding targets and exert pharmacological activities [9]. The essence of the drug molecules binding to the amino acid residue of the receptor is to form a drug-receptor complex and interacts, mainly by the intermolecular electrostatic interaction and ionic bond, hydrogen bond, and Van der Waals forces, etc., thereby activating or inhibiting the bioactivities of the receptors [10,11]. Innovative drug research will be of significant social and economic benefits, and presently, the number of known compounds has reached tens of millions, which provides the basis for drug research and development with big data. At this point, relying on traditional pharmacology and experimental models to test tens of thousands of compounds could be extremely time-consuming and money-consuming.

Target recognition is the first step in modern drug research and development [12], since most of the important physiological processes in organisms, such as cell cycle regulation, anabolism, signal transduction, and transmission of genetic information, closely rely on the interaction and recognition of proteins and ligands [13,14]. Molecular docking is a theoretical method for studying the interaction and recognition between proteins and ligands. By analyzing the interactions between small molecule ligands and receptor biomacromolecules, this method could predict the binding mode and affinity strength, and then realize structure-based drug design, which is of great significance to the molecular mechanisms of pharmacological activities, structure prediction of protein–ligand complexes, and targeted drug screening [15,16,17]. Computer-aided drug design (CADD) is a computational chemistry method for designing and optimizing drug lead compounds by computer simulation, calculation, and prediction of the relationship between drugs and receptors. This method can greatly improve the success rate of drug screening, reduce the blindness of research, exhibit the advantages of low cost and short cycle, and is one of the important means of drug research and development [3]. With the development of structural biology and the improvement of computer performance, forward and reverse molecular docking technologies aroused at the historic moment. It uses computer simulation to place small molecules (ligands) into the binding region of macromolecular targets (receptors), then calculates the physical and chemical parameters to predict the binding force and models of the ligand–receptor complexes, so as to achieve high-throughput, virtual screening of the unknown compounds and to greatly improve the speed of new drug design and discovery [18,19].

Due to the relatively simple operation procedures of molecular docking, and the growing attention on the research of marine drugs, the number of the reports in this field has been increasing in the past 20 years, and the molecular docking technology has become a powerful tool for the research of marine drugs worldwide. To this end, the present review would introduce the basic principles and software of the molecular docking, further summarize the applications of this method in marine drug discovery and design, and finally, discuss and prospect the problems of the molecular docking, in order to provide more theoretical basis for clinical practices and new marine drug research and development.

## 2. Principles of Molecular Docking

### 2.1. Basic Theories 

**Figure 2:**
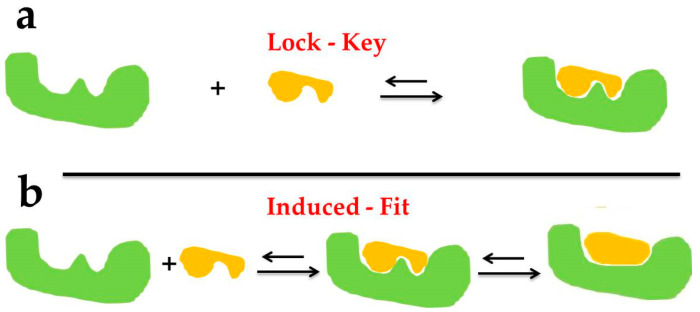
The docking types of Lock–Key Model (**a**) and Induced Fit Theory (**b**).

The Lock–Key Model principle was proposed by Fisher in 1894 and was first applied to explain the theoretical model of the receptor–ligand interaction, which stated that ligands and receptors could recognize each other through geometric matching and energy matching [20]. As shown in Figure 2a below, the ligand enters the receptor in a manner similar to lock and key. At this time, both the receptor and ligand are regarded as rigid structures, that is, the spatial conformation does not change during the molecular docking between the ligand and the receptor. In the Lock–Key Model, the receptor and drug molecules are treated as rigid structures, which can explain well the process of small changes in three-dimensional structure and conformation before and after the binding of the drug and receptor but is slightly insufficient for the larger conformation changes before and after binding.

Considering the limitations in the Lock–Key Model and the enzyme conformation changes due to substrate induction during the enzyme–substrate (ligand) interaction, Koshland proposed the Induced Fit Theory in 1958 [21]. As shown in Figure 2b above, the active site spatial conformation of the protein changes by interaction with the ligand, that is, the protein meets the substrate first and then changes its spatial structure subsequently, which indicates that both ligand and protein are considered as flexible structures during molecular docking. When the Induced Fit Theory was extended to the drug molecule–receptor interaction, it turned out that the docking results obtained by considering ligands and receptors as flexible structures could be more accurate [22].

### 2.2. Molecular Docking Methodologies

#### 2.2.1. Rigid Docking 

In the process of Rigid Docking calculation, the conformation of the ligands and receptors does not change, only the spatial position and posture of the two molecules change [23]. In this kind of docking simulation, the spatial conformation of the ligand and the receptor is regarded as fixed. Namely, this docking method is the most convenient due to the simplest calculation difficulty and calculation amount. Therefore, it is suitable for investigating the docking system with relatively large structures, such as the protein–protein and protein–nucleic acid complexes. In this field, Stoddard et al. treated the ligand and acceptor backbone structures with rigidity, and successfully implemented the docking simulation of maltose and protein by the binary docking method [24].

#### 2.2.2. Flexible Docking

During the flexible docking calculation, the conformation of ligand and receptor is allowed to change freely. Because this kind of docking simulation is of high accuracy, and closest to the real docking situation, it is often used to accurately investigate the recognition between two molecules. However, due to the geometric growth of variables with the number of atoms in the system, the flexible docking method is computationally intensive and time-consuming and requires high requirements on computer software and hardware systems. The most representative molecular docking software is FlexX [25], and Mangoni et al. have used flexible ligands to dock with flexible receptors in this research area previously [26]. 

#### 2.2.3. Semi-Flexible Docking

In the semi-flexible docking calculation process, the conformation of the receptor is rigid and unchanged, and only the conformation of the ligand is allowed to vary within a certain range, such as fixing the bond angle and bond length of some non-critical parts. This docking method has been widely used in the docking simulation between small molecules and biomacromolecules (proteins, enzymes, and nucleic acids) because of its ability of both calculation and prediction of the model [19]. Currently, the commonly used semi-flexible docking programs are FlexX, Dock, AutoDock, etc. [27,28].

### 2.3. Molecular Docking Searching Algorithms

According to classical thermodynamics, the interaction between protein and ligand is a process of systematic thermodynamic equilibrium, and by which the complex structure formed should be the conformation with the lowest binding free energy [29]. Therefore, on the one hand, mathematical models or functions should be employed to calculate the combined free energy as accurately as possible; on the other hand, there is a need to develop effective search algorithms to quickly find conformation with extremely low free energy. 

Considering that the binding between proteins and ligands is a complicated dynamic interaction process at the lowest energy conformations [30], the current conformational searching methods of protein–ligand docking are generally split into three types: exhaustive searching, heuristic searching, and other searching methods, and which in the end, are responsible for calculating a reasonable conformation of the ligand–receptor complexes.

#### 2.3.1. Exhaustive Searching Algorithms

**Figure 3:**
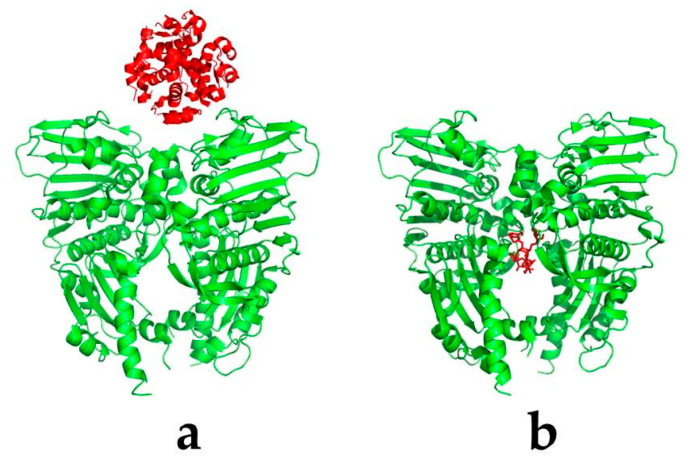
The interaction interfaces of the protein–ligand complexes. The ligand (red) represents the protein (**a**) or small molecule (**b**), respectively. The protein (receptor) is green.

Exhaustive searching is to enumerate various possible situations of the problem one by one when no clear solutions and rules can be found, and select the conditions that meet the requirements as the candidate solutions of the problem through certain evaluation principles [31]. Namely, if the ligand is a biomacromolecule, such as a protein, RNA, or DNA, the interacting region can appear anywhere on the surface of the molecule (Figure 3a), in which a global search algorithm is often required. A typical algorithm is the fast Fourier transform (FFT) algorithm proposed by Kathcalski-Katzir in the protein–protein docking [32]. 

In this method, the protein–ligand complexes are firstly represented as three-dimensional grid data, and the following evaluation principles, such as the degrees of molecular geometry or energy matching, are described quantitatively using correlation functions. The specific evaluation formula can be expressed as:

(1) $$c_{\alpha ,\beta ,\gamma }=\overset{N}{\underset{l=1}{\sum }}\overset{N}{\underset{m=1}{\sum }}\overset{N}{\underset{n=1}{\sum }}a_{l,m,n}•b_{l+\alpha ,\;m+\beta ,n+\gamma }\;$$

In the formula, *a* and *b* are the three-dimensional grid data of protein a and ligand b, respectively, which can represent various grid forms such as geometric, electrostatic, or statistical potential. Among them, *α*, *β*, *γ* are the numbers of grid points where the center of mass of ligand b is shifted relatively to the center of mass of protein a in three directions under the Cartesian system, respectively. Namely, (*α*, *β*, *γ*) is the translation vector of ligand b relative to protein a. *N* is the maximum grid point value of a three-dimensional grid point.

Currently, the FFT algorithm is widely used for fast and exhaustive calculation of geometric matching, electrostatic interactions, and atomic pair preference due to its high efficiency, and this method is commonly employed for most protein–ligand molecule docking programs, such as FT-DOCK [32], 3D-DOCK [33], ClusPro [34], ZDOCK [35], and DOT [36]. 

Besides, similar protein–protein docking programs are the spherical polar Fourier (SPF) transform-based searching algorithm in HEX [37] and FRODOCK [38], along with the advantages that the operation of rotating the ligand can be realized by transforming its expansion coefficient, which greatly reduces the amount of calculation that needs to be cyclically searched for the rotation angle. It is even possible to express the rotational degrees of freedom as coefficients and perform a five-dimensional Fourier transform.

#### 2.3.2. Heuristic Searching Algorithms

On the other hand, if the ligand is a small-molecule compound, the molecular docking often has a certain binding pocket (Figure 3b), which can define the search range of the conformation space, and usually uses an heuristic searching algorithm to search for the defined region. The heuristic searching firstly encodes the translational and rotational operations of ligand molecules in the docking system randomly, then optimizes and selects the ligand conformation after the operation according to the energy score, and finally finds the ligand molecule conformation with the lowest energy. Currently, the representative heuristic searching algorithms in molecular docking are the Monte Carlo (MC) algorithm [39], genetic algorithm (GA) [40], and swarm intelligence (SI) algorithm [41]. 

The MC algorithm first randomly samples in the constellation space, evaluates the sampled function value, selects the value of the function, and retains the obtained optimal solution as the final solution. This method is not affected by the spatial structure and distribution of the research problem and theoretically converges to the global optimal solution when the number of samples approaches infinity. The physical basis of this approach is consistent with molecular docking, that is, looking for lower energy states. Taking a certain conformation of the ligand molecule as the initial state σ, a new state σ’ is generated through random translation and rotation. The energy *E* (*σ’*) and *E* (*σ*) of the two states is judged by calculating the ratio of the Boltzmann factor, and the calculation formula is as follow [39]:

(2) $$r=\exp\left(\frac{E\;\left(\sigma \right)-E\;\left(\sigma^{\prime }\right)}{kT}\right)$$

where *T* is the absolute temperature, and *k* is the Boltzmann constant. As a general searching method, the MC algorithm may require a longer calculation time to give a better solution in practical use. This method now has been adopted in RosettaDock [42] and Glide [43] for full-space conformation searching. The RosettaDock program, in particular, often takes a long time for the bio-macromolecules docking.

The GA algorithm, proposed by Holland in 1975, regards complex optimization problems as the genetic and evolutionary process of organisms [40]. This method first randomly generates the solution of the problem, then performs crossover and mutation operations, continuously optimizes the candidate solutions through fitness selection, and finally converges to the local optimal solution or even the global optimal solution. During the processes, proteins are generally immobile, while the translation and rotation of the ligands relative to the proteins are individual binary data, and the energy function of the protein–ligand interaction is used as the fitness function. That is to say, the conformation with lower energy corresponds to higher individual fitness and will be selected. 

Presently, the GA algorithm has been widely applied in various fields of bioinformatics due to its simplicity, high efficiency, and easy parallel processing and has become one of the most important conformational search algorithms in protein–ligand molecular docking. The representative GA algorithm is the Lamarckian genetic algorithm in the protein–small-molecular docking program AutoDock 4.0 [44] and GOLD [45]. In theory, the heuristic search algorithm can also be applied to the global search algorithm, but this algorithm is more commonly used for the defined region search due to the limitation of calculation time.

The SI algorithm derives from the study of group behavior in nature, where the SI system consists of a group of individuals interacting with each other and with the surrounding environment, and simulates the group self-organizing behavior [41]. Compared with the GA algorithm, the SI algorithm has a higher search efficiency, stronger global optimization ability, and faster convergence speed. Therefore, this method has been quickly introduced into the application of molecular docking conformation search. In this respect, SwarmDock uses the particle swarm algorithm for full-space conformation search [46].

### 2.4. Scoring Functions 

#### 2.4.1. Classifications of Scoring Functions

A successful molecular docking program requires a reasonably sensitive scoring function to rank the complex conformations generated by the searching algorithm in order to pick out near-natural structures. The current scoring functions can be roughly classified into three categories: physics-based scoring function, experience-based scoring function, and knowledge-based scoring function.

For the physics-based scoring function, it refers to the use of the “thermodynamic master equation” for free energy prediction and scoring, specifically based on the force field (such as Amber [47,48] and CHARMM [49]) combined free energy calculation method. This kind of scoring function takes internal energy, solvent effect, and entropy effect into account, and the calculation of combined free energy is relatively accurate, but this calculation procedure is very time-consuming. Generally speaking, the molecular docking programs of GeauxDOCK [50] and GalaxyDock [51] for protein–small molecules contain such a scoring function. 

For the experience-based scoring function, it considers many factors, such as residue pair preference, geometric complementarity and electrostatic, hydrogen bonding, hydrophobic interaction energy, etc. Compared with the physics-based scoring function, the calculation speed of the experience-based scoring function is obviously improved, and thus it has been adopted in many molecular docking programs, including but not limited to FlexX [52], LUDI [53], ZDOCK [54], and RosettaDock [42]. However, this kind of scoring function also has the disadvantages of relying on the decomposition form and the training dataset that produces the weight coefficient. 

For the knowledge-based scoring function, it is obtained by analyzing the existing protein structure database by Boltzmann distribution. Specifically, it is to analyze the complex structure measured in the experiment and obtain the interaction rules. Presently, it is commonly used in the residues–residue contact potential, residue pair preference, and atom–atomic contact potential [55,56]. This type of scoring function is fast and has a high success rate, but it relies too much on the known protein structure data, and also, it is difficult to analyze the specific details during the ligand–protein interaction.

#### 2.4.2. Classic Scoring Function Software

Three types of representative docking software, including ZDOCK, RosettaDock, and high ambiguity driven biomolecular docking (HADDOCK), will be introduced here for their compositions and designs of their scoring functions.

ZDOCK uses the FFT method for rigid docking, and the docking structure uses geometric complementarity, desolvation energy, and electrostatic interaction for rough scoring and screening [54]. In order to evaluate the scoring results more accurately, the subsequent development of ZRANK adopts a more accurate scoring method [57], and the scoring function equation is expressed as:

(3) $$\begin{array}{c} S_{ZDOCK}=W_{vdW_{a}}•E_{vdW_{a}}+W_{vdW_{r}}•E_{vdW_{r}}+W_{elec_{sra}}•E_{elec_{sra}}+W_{elec_{srr}}\\ \;•E_{elec_{srr}}+W_{elec\_lra}•E_{elec\_lra}+W_{elec\_lrr}•E_{elec\_lrr}\\ \;+W_{ds}•E_{ds}\; \end{array}$$

where *E_vdW_a_* and *E_vdW_r_* are Van der Waals attraction and repulsion energy terms, *E_elec_sra_* and *E_elec_srr_* are short-range electrostatic attraction and repulsion energies, *E_elec_lra_* and *E_elec_lrr_* are long-range electrostatic attraction and repulsion energies, and *E_ds_* is desolvation energy, respectively. The corresponding weight parameters are: *W_vdW_a_* = 1.0, *W_vdW_r_* = 0.009, *W_elec_sra_* = 0.31, *W_elec_srr_* = 0.34, *W_elec_lra_* = 0.44, *W_elec_lrr_* = 0.50, *W_ds_* = 1.02. In the later stage of scoring, RDOCK62 can also be used for the further energy optimization of the top 2000 docking structures to eliminate atomic overlap [58]. Similar docking software to ZDOCK includes 3D-Dock [33], DOT [36], BiGGER [59], PatchDock [60], etc.

RosettaDock employs the MC algorithm to optimize the molecular structures, including side chain coating, rigidity minimization, and the final scoring process, and different scoring functions are used for evaluation at different stages [42]. The formula of the scoring function is:

(4) $$\begin{array}{c} S_{RosettaDock}=W_{atr}•E_{atr}+W_{rep}•E_{rep}+W_{sol}•E_{sol}+W_{sasa}•E_{sasa}\\ \;+W_{hb}•E_{hb}+W_{dun}•E_{dun}+W_{pair}•E_{pair}\;+\;W_{elec}^{sr-rep}\\ \;•E_{elec}^{sr-rep}\;+\;W_{elec}^{sr-atr}•E_{elec}^{sr-atr}+\;W_{elec}^{lr-rep}•E_{elec}^{lr-rep}\;\\ \;+W_{elec}^{lr-atr}•E_{elec}^{lr-atr}\; \end{array}$$

where *E_atr_* and *E_re_*_p_ are Van der Waals attraction and repulsion terms, *E_sol_* is the implicit solvation energy, *E_sasa_* is the solvation energy based on surface area, *E_hb_* is the hydrogen bond score, *E_dun_* is the corner probability term, *E_pair_* is the residue pairing potential, $E_{elec}^{sr-rep}$ and $E_{elec}^{sr-atr}$ are short-range electrostatic attraction and repulsion terms, respectively, and $E_{elec}^{lr-rep}$ and $E_{elec}^{lr-atr}$ are long-range electrostatic attraction and repulsion terms, respectively.

HADDOCK combines energy optimization and molecular dynamics simulation for molecular docking [61]. First, a conformation search is performed through rigid energy optimization and semi-flexible simulated annealing, and then molecular dynamics simulation with apparent water is used for further structural improvement. The formula of the scoring function is:

(5) $$S_{HADDOCK}=W_{vdW}•E_{vdW}+W_{elec}•E_{elec}+W_{AIR}•E_{AIR}+W_{BSA}•A_{BSA}+W_{desolv}•E_{desolv}$$

where *E_vdW_* is the Van der Waals term, *E_elec_* is the electrostatic interaction, *E_AIR_* is the fuzzy interaction constraint term, *A_BSA_* is the embedding surface area, and *E_desolv_* is the desolvation energy, respectively. 

The HADDOCK program is characterized by the introduction of site constraint information (i.e., AIR) into the scoring items, as well as structural optimization using precise molecular dynamics simulations with significant water content [62]. Due to the consideration of the influence of experimental information and water in the process, in the docking test determined by experimental information, the convergence to the correct structure can be rapidly achieved, and the accurate composite structure can be obtained [63]. However, if experimental information is lacking, the scoring effect will be affected.

### 2.5. Molecular Docking Softwares

After decades of development and application, especially the promotion of drug research and development, a considerable number of molecular docking programs have been developed all over the world. Most of them are software for docking small molecules (ligand) and proteins (receptor), and software for docking protein–protein, protein–DNA, and protein–RNA molecules. This docking software was originally developed by laboratories and released for free. When some software is upgraded with very limited defects, it may be purchased by a specialized commercial software company and becomes a module in a large software package. Table 1 below lists some of the commonly used molecular docking programs and summarizes their algorithm characteristics and applications. In these applications, for example, freely available software mainly include DOCK, AutoDock, AutoDOCK Vina, 3D-DOCK, LeDock, rDock, UCSF DOCK, Surflex (for academic users), and HEX; while commercial software consists mostly of Glide, GOLD, MOE Dock, ICM-Dock, MCDOCK, Surflex-Dock, LigandFit, FlexX, and so on [64]. 

## 3. Applications of the Molecular Docking in the Research and Discovery of Potential Marine Drugs

In recent decades, a great number of marine-derived active compounds have been discovered and studied worldwide, many of which have been approved for marketing or entered different clinical research stages. The metabolites of marine organisms are not only structurally diverse and novel but also have strong biological activity, which provides a large number of model structures and pro-drugs for the research and development of new drugs. Molecular docking technology, as a major method of computer-aided drug design, has been widely applied in screening active components and elucidating the mechanisms of action and played an important role in marine drug research and development in recent decades.

### 3.1. Target Proteins of Melanin Formation

**Figure 4:**
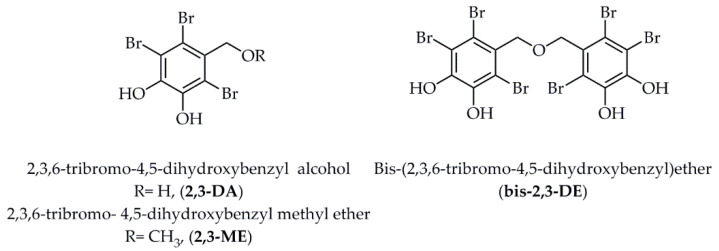
Structures of three halogenated compounds isolated from marine algae *Symphyocladia latiuscula*. Abbreviations: 2,3-DA, 2,3,6-tribromo-4,5-dihydroxybenzyl alcohol; 2,3-ME, 2,3,6-tribromo-4,5- dihydroxybenzyl methyl ether; bis-2,3-DE, bis-(2,3,6-tribromo-4,5-dihydroxybenzyl)ether.

Tyrosinase is a kind of copper-containing metal oxidase that regulates the melanin production [79]. Three natural halogenated compounds of 2,3-DA (2,3,6-tribromo-4,5-dihydroxybenzyl alcohol), 2,3-ME (2,3,6-tribromo-4,5-dihydroxybenzyl methyl ether), and bis-2,3-DE (bis-(2,3,6-tribromo-4,5-dihydroxybenzyl)ether) (Figure 4), isolated from the nutrient-rich marine algae *Symphyocladia latiuscula* [80], exhibited potential inhibition on mushroom tyrosinase with the IC_50_ values at 10.78 ± 0.04 µM, 113.94 ± 0.75 µM, and 2.92 ± 0.04 µM, respectively. For the further prediction of the binding sites, the molecular docking method was employed and indicated that the most potential bromophenol, bis-2,3-DE, formed two hydrogen bonds with the amino acid residues of Arg 268 and Per 404 (peroxide ions), with the lowest binding energy of −7.81 kcal/mol. Meanwhile, 2,3-DA formed three hydrogen bonds with the amino acid residues of Asn 260, His 61, and Per 404; 2,3-ME only formed one hydrogen bond with the amino acid residue of Per 404, with the binding energies at −6.19 kcal/mol and −6.29 kcal/mol, respectively. Hence, it was assumed that the catalytic hydrogen and halogen interactions between the three halogenated compounds and tyrosinase residues could be responsible for the anti-tyrosinase activity.

### 3.2. Target Proteins of Diabetes Mellitus

For some of the compounds mentioned above in Figure 4, Paudel et al. [81] revealed that the bromophenol, bis-2,3-DE, exerted most potential inhibitory effects against tyrosine phosphatase 1B (PTP1B) and α-glucosidase, with the IC_50_ values of 5.29 ± 0.08 µM and 1.92 ± 0.02 µM, respectively. Additionally, the bromophenol bis-2,3-ME formed three hydrogen bonds in the active catalytic pocket with the amino acid residues of PTP1B at Lys 116, Arg 221, and Cys 215 with the binding energy of −6.86 kcal/mol. Likewise, this bioactive compound formed three hydrogen bonds in both catalytic and allosteric regions with α-glucosidase at the amino acid residues of Gln 353, Ser 157, and Asp 307 with the binding energy of −8.06 kcal/mol. Later, the importance of the 7-OH group for hydrogen bond formation and the importance of bromine/benzene ring numbers for halogen bond interaction were proved in the molecular docking simulation.

**Figure 5:**
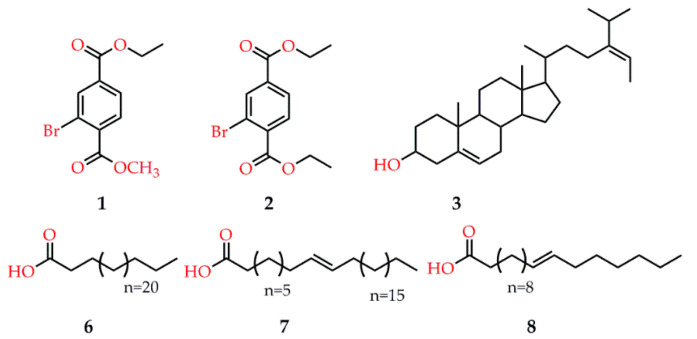
Potential compounds isolated from the seaweeds of *Dictyopteris hoytii*.

In another study, two new natural brominated and six known metabolites were isolated from marine macro brown alga *Dictyopteris hoytii* (Figure 5), among which compound **7** displayed the highest inhibition against α-glucosidase with the IC_50_ at 30.5 ± 0.41 µM, followed by compounds **2** and **3** with the IC_50_ values at 234.2 ± 4.18 and 289.4 ± 4.91 µM, respectively. Molecular docking simulations of the binding mode revealed that the acetate -OH group of compound **7**, on the one hand, served as H-bond donor to the side chain of Asp 214 to destabilize the catalytic triad for further action and, on the other hand, accepted H-bond from the side chain of His 111 to further strengthen binding of this ligand within the active site. Similarly, the ethyl ester moiety of compound **2** interacted with the side chain of Arg 439 and a water molecule (Wat1174) via H-bond; while the -OH group of compound **3** acted as H-bond acceptor to the side chain of His111 and mediated the bidentate interactions with the side chains of Asp214, suggesting that the acetate moiety of these bioactive compounds was essential for binding to the catalytic residues [82]. Interestingly, the positive control of acarbose also exhibited strong interactions with the active sites of α-glucosidase because of its high number of -OH moieties.

### 3.3. Target Proteins of Hypertension

A large number of by-products are produced during the processing of tilapia fillets. For example, about 80% of the protein in fish bones and skin is underutilized [83]. Studies have shown that two proteins in tilapia bone and skin exhibited good blood pressure lowering activity, which may be precursors of antihypertensive peptides [84]. Furthermore, other bioactive peptides, such as MetVal-Gly-Ser-Ala-Pro-Gly-Val-Leu (MVGSAPGVL) from skate skin gelatin [85], and Thr-Gly-Gly-Gly-Asn-Val (TGGGNV) from Pacific cod skin gelatin [86], have also been revealed to exhibited arresting antihypertensive effects.

In addition, Leu-Trp-His-Thr-His (LWHTH), an antioxidant peptide purified from *Styela clava*, displayed the maximum reduction of 89.4% on the systolic blood pressure (SBP), and 83.8% on the diastolic blood pressure (DBP) of rats at 3 h, respectively, after the single oral administration at 40 mg/kg of body weight. Meanwhile, this peptide exerted noteworthy inhibition on angiotensin I-converting enzyme (ACE) with the IC_50_ at 16.42 ± 0.45 µM in a concentration-dependent manner. The following interaction simulations between LWHTH and ACE using CDOCKER tool indicated that 2 H-bonds to Arg 522 and Glu 403, and a π H-bond to Lys 118 formed a network at the binding position of the LWHTH-ACE complex; besides, the hydrophilic tripeptide HTH was fitted to the dished surface of the active site, which revealed the importance of this tripeptide sequence to LWHTH for its inhibitory effects. Finally, the stability of the LWHTH–ACE complex was further verified with low score values of CDOCKER interaction energy (the H-bonds from leucine and tryptophan) at −102.566 kcal/mol, and the total binding energy at −372.069 kcal/mol. In this regard, LWHTH strongly inhibited the biological function of ACE, so as to display notable antihypertensive effect [87].

### 3.4. Target Proteins of Inflammation

**Figure 6:**
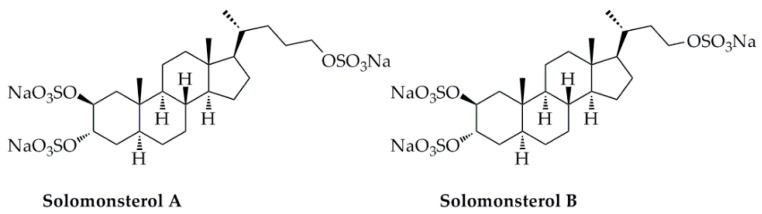
Two sulfated sterol derivatives (solomonsterols A, B) isolated from *Theonella swinhoei*.

Pregnane X Receptor (PXR) is a member of the nuclear receptors (NRs) superfamily and exerts immunomodulatory and anti-inflammatory activities by inhibiting the function of NF-κB [88]. Solomonsterol A, a selective PXR agonist, along with its other sulfated sterol solomonsterols B (Figure 6), extracted from the marine sponge *Theonella swinhoei*, exerted anti-inflammatory activity and attenuates systemic inflammation and immune dysfunction in a mouse model of rheumatoid arthritis, and the further structure-function relationship revealed that a truncated C24 side chain and three sulfate groups at C2, C3, and C24 were the key functional groups of solomonsterol A [89]. In addition, the molecular docking simulations were employed to examine the positions in the binding sites of PXR using the AutoDock 4.2 software. The calculation results revealed that the three sulfate groups of solomonsterol A closely interacted with the amino acids of Ser 247, His 407, and finally with Lys 210, and contributed to accommodating the steroid nucleus in a mostly hydrophobic part of the binding site of PXR. To be specific, solomonsterol A formed two H-bonds with Cys 284 (2-*O*-sulfate) and Lys 210 (24-*O*-sulfate), and electrostatic interactions with Ser 247 (2-*O*-sulfate) and His 407 (3-*O*-sulfate). Therefore, it was demonstrated that the three sulfate groups in solomonsterol A contributed to accommodate the steroid nucleus in PXR-LBD by acting as key points of interactions with those three polar amino acids [90].

**Figure 7:**
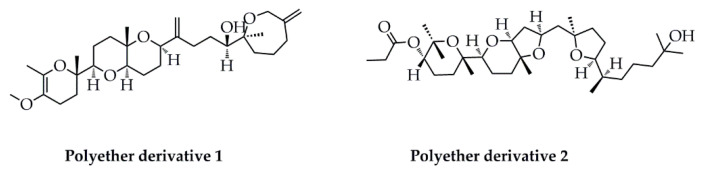
Two polyether derivatives (compounds **1**, **2**) isolated from the thalli of *Gracilaria salicornia*.

Cyclooxygenase-2 (COX-2) and 5-lipoxygenase (5-LOX) are the two key rate-limiting enzymes in the development of pro-inflammatory prostaglandins/thromboxanes by COX-2 and leukotrienes by 5-LOX pathways [91], respectively. In the ongoing research for selective COX-2/5-LOX inhibitors with anti-inflammatory properties from marine natural products [92], compound **2** (Figure 7), isolated from the thalli of marine macroalga *Gracilaria salicornia*, effectively attenuated COX-2/5-LOX enzymes, along with retaining COX-2/COX-1 ratio within the threshold limits (IC_50_ of anti-COX-2 to IC_50_ of anti-COX-1 < 1) for selective and target-oriented potencies against inflammatory response, compared to that displayed by compound **1**. Furthermore, in silico docking modelling studies against COX-2 and 5-LOX revealed that compound **2**, bearing furanyl-furo [3,2-b] pyran-2H-pyran moiety, formed 4 H-bond interactions with the amino-acyl side chains of COX-2 at Ser 144 (2 bonds), Ser 147, and Asn 145 in the enzyme active zone with molecular distances of 3.184/3.016, 3.217, and 3.152 Å, respectively. Meanwhile, compound **2** also exhibited 2 H-bond interactions with the amino-acyl units of 5-LOX at the Arg 246 and Asp 442 in the active site of enzyme with molecular distances of 2.856 and 3.266 Å, respectively. In addition, compound **2** exerted closer molecular associations to COX-2 and 5-LOX with minimum binding energy of −10.29 kcal/mol and −10.96 kcal/mol, and intermolecular energy of −11.73 kcal/mol and −12.23 kcal/mol, respectively. Conclusively, those aforementioned results not only strongly demonstrated the great potential of compound **2** for future clinic usage against inflammatory pathophysiologies but also highlighted the importance of the furanyl-furo [3,2-b] pyran-2H-pyran skeleton in the polyether triterpenoid as the potential pharmacophore lead for the discovery and development of anti-inflammatory drugs.

### 3.5. Target Proteins of Severe Acute Respiratory Syndrome Coronavirus 2 (SARS-CoV-2)

The worldwide spread of the SARS-CoV-2 has posed a great threat to global public health in 2020. Previous studies suggested that the main protease (M^pro^) of SARS-CoV-2, sharing a 96% similarity of sequence alignment to that of SARS-CoV-1 [93], acted as a highly validated drug target for the treatment of COVID-19 [94]. To this regard, Gentile et al. carried out a virtual screening of a library of 14,064 marine natural products for searching new, potential SARS-CoV-2 M^pro^ inhibitors [95]. The compound library was first screened with a hyphenated pharmacophore model, wherein 180 compounds were further docked with AutoDock Vina, along with a parallel docking study with AutoDock4 and molecular dynamics simulations. In this result, 17 compounds, belonging to a class of phlorotannins, oligomers of phloroglucinol isolated from *Sargassum spinuligerum* brown alga, exerted the most potential inhibition on the M^pro^ of SARS-CoV-2. Among them, heptafuhalol A exhibited the lowest docking energy of −14.60 kcal/mol. Docking mode showed that the –OH groups of this compound served as the H-bond donors to the protease residues of Thr24, Ser46, Asn142, Glu166, and Pro168. Meanwhile, both the π H-bonds of this compound to the side chains of His41 and Gly143 and the hydrophobic interactions with the residues of Met49, Met65, Leu141, and Pro168 also contributed to the stabilization of the ligand–receptor complexes. Finally, the screened 17 compounds exhibited higher energy scores than the current drug used for the treatment of COVID-19 and hence were validated as potential SARS-CoV-2 M^pro^ inhibitors.

### 3.6. Target Proteins of Cancer

**Figure 8:**
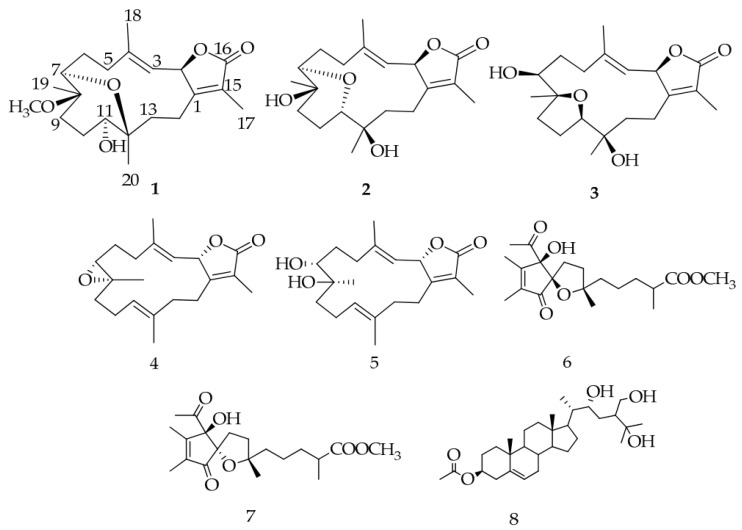
Chemical structures of metabolites **1**–**8** isolated from the soft coral *Sarcophyton ehrenbergi*.

In an effort to find soft coral metabolites in the Red Sea, eight compounds were isolated from the *Sarcophyton ehrenbergi* (Figure 8), in which one steroid, sardisterol, displayed significant inhibition on A549 cancer line with the IC_50_ value at 27.3 µM, followed by the Hep G2 cell lines with the IC_50_ value at 56.8 µM [96]. For the first time, sardisterol was proven to be a potent anticancer candidate in this regard. Considering that overexpressed the epidermal growth factor receptor (EGFR) correlated closely with several non-small-cell lung carcinomas [97], the EGFR kinase was employed to be the target for the anti-proliferative activity of sardisterol against A549 [98]. The molecular docking results stated that sardisterol interacted with EGFR by forming two hydrogen bonds with the active sites of Thr 766 and Asp 776, and the average hydrogen bond length between sardisterol and Asp 776 was 2.16 Å. As a consequence, the molecular docking studies correlated with the inhibition of the cancer cell growth.

**Figure 9:**
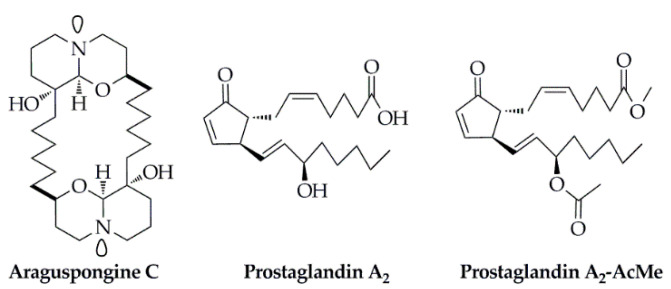
Structures of the small molecule compounds isolated from the marine sponge *Xestospongia exigua* (Araguspongine C) and the octocoral *Plexaura homomalla* (Prostaglandin A_2_ and Prostaglandin A_2_-AcMe), respectively.

Araguspongine C (Figure 9), an oxaquinolizidine alkaloid isolated from the marine sponge *Xestospongia exigua*, not only displayed remarkable anti-proliferative activities against six breast cancer cell lines but also induced distinct autophagic death of BT-474 cells at 10 μM [99]. Besides, for the interpretation of the occurrence of cellular autophagy, araguspongine C suppressed the phosphorylation of the c-Met in vitro, one of the key receptor tyrosine kinase in the development of the breast cancer, with the IC_50_ value at 19.9 μM in a dose-dependent manner. For the molecular docking studies, on the one hand, araguspongine C was partially wrapped around the Met 1211 at the activation loop of the c-Met kinase domain with a shallow U-shaped binding pattern. The C-9′ hydroxyl group of the oxaquinazoline ring and the side chain phenolic hydroxyl group of Tyr 1159 of the hinge region were both involved in critical single-point hydrogen bonding interactions. In addition, the hexa-carbon aliphatic linker on the dimeric oxaquinolinazine ring system was hydrophobic with the side chains of Ile 1084, Val 1092, Ala 1108, and Leu 1140 on the hydrophobic sub-pocket of the c-Met kinase domain. On the other hand, araguspongine C interacted with the HER2, another highly expressed receptor tyrosine kinase during the autophagy BT-474 of breast cancer, through a hydrogen bond between the C-9′ hydroxyl group and the carboxylate side chain of Asp 863. Considering the close relationship between the suppression of the c-Met and HER2 and the cellular autophagy, the C-9′ hydroxyl group of araguspongine C was supposed to be the crucial pharmacophoric group for its notable anti-proliferative activities against breast cancer cell lines and in vitro enzyme inhibition activities, compared with another oxaquinolizidine alkaloid of araguspongine A without the C-9′ hydroxyl group.

In another study, two prostaglandin derivatives, prostaglandin A_2_ and prostaglandin A_2_-AcMe (Figure 9), were isolated from the octocoral *Plexaura homomalla* [100]. Prostaglandin A_2_ displayed the most potential inhibitory effects on human breast cancer cell line (MDA-MB-231) and lung cancer cell line (A459) with the IC_50_ values at 16.46 μg/mL and 25.20 μg/mL, respectively. Additionally, this compound inhibited the enzyme p38α kinase and non-receptor tyrosine kinase (c-Src) by 49% and 59% at a concentration of 2.5 μM, while prostaglandin A_2_-AcMe generated 42% and ≤ 40% inhibition, respectively; meanwhile, prostaglandin A_2_ displayed 64% inhibition on topoisomerase IIα at a concentration of 10 μM, compared with the positive control of doxorubicin of 88% inhibition. For the further molecular docking studies, the binding mode (through interactions) and affinity (as Vina scores) were determined using AutoDock/Vina. The docking results revealed the binding scores of all compounds above −8.0 kcal/mol. For the case of p38α-kinase, the best interaction occurred with derivative 5 forming three H-bonds with Lys 53 and Glu 71. However, for the other two enzymes, the best interaction was generated with the natural prostaglandin A_2_. In the case of topoisomerase IIα, 18 H-bonds were formed with Asn 150, Lys 157, Ser 149, Thr 147, Lys 168, Ala 167, Leu 169, Asn 120, Thr 181, Thr 215, Thr 147, Gln 316, Gln 122, and Lys 123, whereas the Src-kinase–prostaglandin A_2_ complex produced six H-bonds with Ans 131, Asp 404, Phe 405, Gly 406, and Ala 390, respectively. Therefore, results suggested that prostaglandin A_2_ might be considered as an important lead for further studies in cancer research.

## 4. Conclusions and Outlooks

Currently, except for the above-mentioned marine drugs that have been approved for clinical usage, there are many marine drug candidates that have been approved by several drug regulatory agencies worldwide for clinical studies at various stages; for example, Salinosporamide A (Marizomib/NPI-0052) for the treatment of malignant gliomas [101]; Tetrodotoxin (TTX) [102], a non-addictive analgesic for the treatment of advanced cancer, neuralgia, and vasculitis; Plitidepsin (Aplidine) for the treatment of multiple bone marrow [103]; and ASG-5ME for the treatment of pancreatic cancer [104], etc. There are numerous kinds of marine organisms in the ocean, which thereupon produce countless special secondary metabolites. However, the current discovery of the active components in marine organisms is still at the beginning, and less than 1% of the total marine organisms have been systematically studied for their bioactive chemical compositions and corresponding mechanisms of action.

With the development of computer simulation technologies, molecular-docking-based technologies have become the direct methods to discover potential drug targets efficiently and on a large scale. At this point, the advantage of the molecular docking method is that all the molecules in the compound database are known compounds, and a considerable part of them can be easily purchased or synthesized according to the known synthetic route, and so the subsequent pharmacological tests can be conducted quickly. Moreover, it can simulate drug-receptor interactions, elucidate the mechanism of action of drugs, and increase accuracy, sensitivity, specificity, and predictability, which provides a good tool for drug research and development. In recent years, the development of computer technology, the rapid growth of target enzyme crystal structure data and algorithms, and the continuous updating of commercial small molecule databases have made molecular docking a huge success in drug design. However, there are still some common problems in molecular docking, which should be given full attention, such as: first, there are still differences between the virtual data obtained by molecular docking and the experimental data in vivo, and it needs to be verified in combination with other experimental methods. Second, due to the complex and diverse nature of components in marine organisms, it is still necessary to speed up the update of the database. Third, existing molecular docking software programs are cumbersome and complex, and they need to be optimized. Fourth, the existing evaluation methods for molecular docking technology are still immature, and molecules with higher scores may not be the best ligands.

Molecular docking technology is the main method of computer-aided drug design (CADD), and with the rapid rise in genomics, proteomics, metabolomics, and other omics technologies, as well as the mutual integration of various subject areas, this technology will help to explore the bioactive substances in marine organisms and the mechanisms of action for treating diseases and promote the research process and cycle of marine natural products.

## Figures and Tables

**Table 1:** Some representative molecular docking programs, and their algorithm characteristics and applications.

Program Name	Algorithm Characteristics	Typical Applications	Ref.
DOCK	Step-by-step geometric matching strategy; AMBER force field experience-based scoring function. As a kind of commonly used molecular docking software, it can be used for docking between flexible small-molecule ligands and flexible proteins.	Protein–small molecule	[65]
AutoDock	Lamarck genetic algorithm and experience-based scoring function; the flexibilities of small molecules and some residue side chains can be fully taken into consideration.	Protein–small molecule	[66]
AutoDock Vina	The upgraded version of AutoDock; the success rate and calculation speed are greatly improved compared to AutoDock; simple parameter setting, easy to use, and parallel operation on multi-core machines for docking flexible ligands and flexible protein side chains.	Protein–small molecule	[67]
MDock	Using the knowledge-based atomic–atomic contact potential scoring function, the flexibilities of proteins and small molecules are considered by using the conformations of the multiple proteins and small molecules during the docking process.	Protein–small molecule	[68]
FlexX	The best conformation is selected according to the size of the docking free energy, which has the advantages of fast speed, high efficiency, and easy operation. It is the representative software of the flexible docking and can also be used for the virtual screening of small molecule database.	Protein–small molecule	[25,52]
GOLD	Based on the GA docking program, the ligand is completely flexible, the receptor binding position is partially flexible; the automatic docking program can be used for virtual screening of the database. Its accuracy and reliability are highly evaluated in the molecular docking simulation.	Protein–small molecule	[45]
Surflex-Dock	The Hammerhead scoring function is used; it combines a large number of conformations from the intact molecules through a crossover process to achieve flexible docking.	Protein–small molecule	[69]
eHiTS	An accurate and fast molecular docking program, which can be used to study ligand and receptor interactions and perform high-throughput virtual screening.	Protein–small molecule	[70]
EADock	Multi-objective evolutionary optimization algorithm for docking small molecules with the active sites of proteins.	Protein–small molecule	[71]
Glide	Docking program based on search algorithms, including the modes of extra precision (XP), standard precision (SP), and a high-throughput virtual filter. It is mainly used for the flexible docking of small-molecule ligands and proteins.	Protein–small molecule	[43]
PIPER	FFT search algorithm; the knowledge-based atomic statistical potential scoring function, and applied to the ClusProServer	Protein–protein	[72]
ZDOCK	FFT search algorithm; filtering and sorting with RDOCK.	Protein–protein	[54]
Hammerhead	Fragment-based docking program for automated and rapid molecular docking of flexible ligands; the program uses an experience-based adjustment scoring function and a method to automatically identify and describe protein binding sites for molecular docking.	Protein–protein/small molecule	[73]
MOE	A comprehensive software system for the pharmaceutical and life science, which could fully support drug design and research through molecular simulation, protein structure analysis, small molecule database processing and protein and small-molecule docking research in a unified operating environment.	Protein–protein/small molecule	[74]
FLIPDock	A genetic algorithm-based docking program that uses the FlexTree data structure to represent the protein–ligand complex and enables docking of flexible ligands and flexible proteins.	Protein–protein/small molecule	[75]
ICM-Dock	User-friendly interactive image display, and the software also supports fast and accurate docking optimization.	Protein–protein/polypeptide/small molecule	[76]
HADDOCK	Docking program based on experimental data (such as NMR chemical shifts and point mutations), which was invented from protein–protein docking and can also be used for protein–ligand docking.	Protein–protein/DNA/RNA/small molecule	[61]
RosettaDock	MC search algorithm; the experience-based energy scoring function.	Protein–protein/DNA/RNA/small molecule	[42]
DOT	FFT search algorithm; the scoring function only has Van der Waals and electrostatic terms.	Protein–protein/DNA/RNA	[36]
FLOG	Rigid docking program using a pre-generated conformation database	Protein–protein/DNA/RNA	[77]
MS-Dock	The method consists of two main steps: first, generate a variety of 3D conformations; second, carry out the rigid docking of the conformations and multi-step virtual screening.	Protein–protein/DNA/RNA	[78]

Abbreviations: Ref., Reference; FFT, fast Fourier transform; GA, genetic algorithm; MC, Monte Carlo; MOE, molecular operating environment; GOLD, genetic optimisation for ligand docking; eHiTS, electronic high-throughput screening; EADock, evolutionary algorithm for docking; FLIPDock, flexible ligand–protein docking; ICM-Dock, internal coordinate modeling docking; HADDOCK, high ambiguity driven biomolecular docking; ZDOCK, Zhiping Weng docking; DOT, daughter of TURNIP; FLOG, flexible ligands oriented on Grid; MS-Dock, multi-stage Dock.
